# Discordance between Liver Biopsy and FibroScan® in Assessing Liver Fibrosis in Chronic Hepatitis B: Risk Factors and Influence of Necroinflammation

**DOI:** 10.1371/journal.pone.0032233

**Published:** 2012-02-23

**Authors:** Seung Up Kim, Ja Kyung Kim, Young Nyun Park, Kwang-Hyub Han

**Affiliations:** 1 Department of Internal Medicine, Yonsei University College of Medicine, Seoul, Korea; 2 Institute of Gastroenterology, Yonsei University College of Medicine, Seoul, Korea; 3 Department of Pathology, Yonsei University College of Medicine, Seoul, Korea; 4 Center for Chronic Metabolic Disease, Yonsei University College of Medicine, Seoul, Korea; 5 Liver Cirrhosis Clinical Research Center, Seoul, Korea; 6 Brain Korea 21 Project for Medical Science, Seoul, Korea; The University of Hong Kong, Hong Kong

## Abstract

**Background:**

Few studies have investigated predictors of discordance between liver biopsy (LB) and liver stiffness measurement (LSM) using FibroScan®. We assessed predictors of discordance between LB and LSM in chronic hepatitis B (CHB) and investigated the effects of necroinflammatory activity.

**Methods:**

In total, 150 patients (107 men, 43 women) were prospectively enrolled. Only LSM with ≥10 valid measurements was considered reliable. Liver fibrosis was evaluated using the Laennec system. LB specimens <15 mm in length were considered ineligible. Reference cutoff LSM values to determine discordance were calculated from our cohort (6.0 kPa for ≥F2, 7.5 kPa for ≥F3, and 9.4 kPa for F4).

**Results:**

A discordance, defined as a discordance of at least two stages between LB and LSM, was identified in 21 (14.0%) patients. In multivariate analyses, fibrosis stages F3–4 and F4 showed independent negative associations with discordance (*P* = 0.002; hazard ratio [HR], 0.073; 95% confidence interval [CI], 0.014–0.390 for F3–4 and *P* = 0.014; HR, 0.067; 95% CI, 0.008–0.574 for F4). LSM values were not significantly different between maximal activity grades 1–2 and 3–4 in F1 and F2 fibrosis stages, whereas LSM values were significantly higher in maximal activity grade 3–4 than 1–2 in F3 and F4 fibrosis stage (median 8.6 *vs*. 11.3 kPa in F3, *P* = 0.049; median 11.9 *vs*. 19.2 kPa in F4, *P* = 0.009).

**Conclusion:**

Advanced fibrosis stage (F3–4) or cirrhosis (F4) showed a negative correlation with discordance between LB and LSM in patients with CHB, and maximal activity grade 3–4 significantly influenced LSM values in F3 and F4.

## Introduction

Because the prognosis of and management strategies for patients with chronic liver diseases depend strongly on the severity of liver fibrosis, early detection of significant fibrosis is key [1]. To date, liver biopsy (LB) has been the gold standard for assessing liver fibrosis. However, its invasiveness, potential adverse events [2], sampling errors [3], and interpretational variability [4] have encouraged clinicians to seek more accurate and noninvasive tools for assessing liver fibrosis.

Recently, liver stiffness measurement (LSM) using FibroScan® was introduced as a noninvasive device to accurately assess liver fibrosis [5]. In view of the results achieved so far, LSM can help physicians decide treatment strategies, predict prognosis, and monitor disease progression or regression in patients with chronic liver disease. Despite the clinical usefulness of LSM, several confounding factors that can diminish the accuracy of LSM have been identified, such as necroinflammatory activity, reflected by a high alanine aminotransferase (ALT) level, cholestasis, or heart failure [6]–[12]. In addition to these extrinsic factors, LSM should satisfy the intrinsic prerequisites for preserving the validity of LSM: ≥10 valid measurements, a success rate ≥60%, and an interquartile range (IQR)/median LSM value among valid measurements (IQR/M) <0.3. However, because these criteria are not based on scientific evidence, several studies have tried to demonstrate the clinical relevance of these criteria by identifying factors that predict discordant results between LB and LSM in estimating liver fibrosis. Results from these studies have identified elevated ALT, high IQR/M, high body mass index (BMI), and fibrosis stage at the time of LB as predictors of discordance [13]–[15]. Although elevated ALT has been considered to be the single most important confounder on LSM, the effects of necroinflammatory activity, which is closely related to ALT level, on discordance between LB and LSM have not been determined.

Thus, in this study, we examined predictors of discordance between LB and LSM in patients with chronic hepatitis B (CHB) and investigated the effects of necroinflammatory activity on LSM.

## Methods

### Patients

Between January 2007 and December 2009, 196 consecutive patients with CHB, defined by detectable hepatitis B virus surface antigen (HBsAg) for more than 6 months and positive hepatitis B virus (HBV) DNA by polymerase chain reaction assay, underwent both LB and LSM before starting antiviral treatment. Of them, 184 (93.9%) patients received LSMs on the same day as LB. The remaining LSMs were conducted at a median of 6 (range, 1–24) days before LB in 12 (6.1%) patients.

No patient had evidence of decompensated liver cirrhosis, such as a history of variceal bleeding, ascitic decompensation, hepatic encephalopathy, or Child-Pugh class B or C at the time of LB and LSM. Exclusions criteria were as follows: (1) previous antiviral treatment before LB, (2) evidence of liver cancer or another malignancy, (3) coinfection wtih hepatitis C virus, hepatitis D virus, or human immunodeficiency virus, (4) alcohol consumption in excess of 40 g/day for more than 5 years, (5) LB specimens shorter than 15 mm in length or unknown LB length, (6) right-sided heart failure, (7) LSM failure, or (8) unrelaible LSM (<10 valid measurments).

This study cohort includes a subset of a previous multicenter Korean study [14]. The study protocol was consistent with the ethical guidelines of the 1975 Declaration of Helsinki. Written informed consent was obtained from each participant or responsible family members after the possible complications of LB had been fully explained. This study was approved by the independent institutional review boards of Severance Hospital, Yonsei University College of Medicine.

### Clinical data

Demographic details and BMI were collected. The following laboratory parameters were also collected from all the patients at the time of LSM; ALT, gamma-glutamyltranspeptidase (GGT), and platelet count. HBsAg was measured using standard enzyme-linked immunosorbent assays (Abbott Diagnostics, Abbott Park, IL, USA). The upper limit of normal (ULN) for ALT was defined as 40 IU/L.

### Liver stiffness measurement

LSM was obtained according to the instructions provided by the manufacturer. Details of the technical background and examination procedure have been described previously [5], [16]–[18]. The success rate was calculated as the number of valid measurements divided by the total number of measurements. Results are expressed in kilopascals (kPa). IQR was defined as an index of LSM intrinsic variability corresponding to the 25^th^ and 75^th^ percentiles intervals around the LSM result containing 50% of the valid measurements. The median value was considered representative of the elastic modulus of the liver. Only procedures with ≥10 validated measurements were considered reliable, regardless of success rate and IQR/M. The same experienced operator (>3,000 LSM examinations), blinded to LB results and the clinical data of the study population performed all LSM examinations.

### Liver biopsy and histological evaluation

LB specimens were fixed in formalin and embedded in paraffin. Sections (4 µm) were stained with hematoxylin and eosin and Masson's trichrome. All liver tissue samples were evaluated by an experienced hepatopathologist (YN Park) who was blinded to the clinical data of study population, including LSM results. Liver fibrosis and necroinflammation were evaluated semiquantitatively according to the Laennec system [19]. Fibrosis was scored in five grades at first: 0, no definite fibrosis, 1, minimal fibrosis (no septa or rare thin septum; may have portal expansion or mild sinusoidal fibrosis), 2, mild fibrosis (occasional thin septa), 3, moderate fibrosis (moderate thin septa; up to incomplete cirrhosis), and 4, liver cirrhosis. Then, liver cirrhosis was sub-classified into three groups: F4A, mild cirrhosis, definite or probable, 4B, moderate cirrhosis (at least two broad septa), and 4C, severe cirrhosis (at least one very broad septum or many minute nodules). The activity grade referred to the degree of necroinflammatory activity in the lobule and periportal area and was scored in five grades: A0, no activity, A1, minimal, A2, mild, A3, moderate, and A4, severe activity. Maximal activity grade was defined as the higher of the lobular and periportal activity. Steatosis in the liver specimen was graded on a four-point scale: S0 (insignificant, <5%), S1 (mild, 5–33%), S2 (moderate, 34–66%), and S3 (severe, ≥66% of hepatocytes with fat deposits) [20], [21].

### Statistical analysis

Patient characteristics are reported as means ± standard deviations, medians (ranges), or *n* (%), as appropriate. Continuous variables of patients with discordance and those without were compared with independent *t-*tests or Mann-Whitney *U* tests. The chi-squared or Fisher's exact test was used for categorical variables. A discordance was defined as a discordance of at least two stages between LB and LSM [13]. Cutoff LSM values for determining discordance were derived from our cohort, which maximized the sum of sensitivity (Se) and specificity (Sp). Positive and negative predictive value (PPV and NPV) was also computed. Spearman's analysis was used to investigate correlations between variables. Univariate and subsequent multivariate binary logistic regression analyses were performed to identify independent factors related to discordance between LB and LSM. Hazard ratios (HRs) and corresponding 95% confidence intervals (CIs) are also indicated. A two-sided *P* value of <0.05 was considered significant. All statistical analyses were performed with the SPSS software (ver. 12.0; SPSS Inc., Chicago, IL, USA).

## Results

### Baseline characteristics of the study population

After excluding 46 patients according to the predefined excludion criteria, 150 were included in the final analysis. Baseline characteristics of the excluded 46 patients including the percentage of discordance, were not significantly different from those of the remaining 150 patients (all *P*>0.05). The baseline characteristics of study population are listed in **Table 1**. The mean age, ALT level, and LSM values were 41.9 years, 74.1 IU/L, and 11.7 kPa, respectively. The proportions of patients with F2–4, F3–4, and F4 were 84.7% (*n* = 127), 56.0% (*n* = 84), and 45.3% (*n* = 68) and those of maximal A2–4, A3–4, and A4 were 88.0% (*n* = 132), 40.0% (*n* = 60), and 12.0% (*n* = 18), respectively. Among patients with cirrhosis (*n* = 68), 8 (11.8%), 44 (64.7%), and 16 (23.5%) patients showed F4A, F4B, and F4C, respectively.

**Table 1:** Baseline characteristics.

	All patients	Patients with non-discordance	Patients with discordance	*P* value
	(n = 150)	(n = 129, 86.0%)	(n = 21, 14.0%)	
Age, years	41.9±14.2	43.1±13.4	34.3±16.7	0.008*
Male	107 (71.3)	93 (72.1)	14 (66.7)	0.610
BMI, kg/m^2^	23.2±2.8	23.1±2.7	23.8±3.5	0.321
Obesity (BMI ≥30 kg/m^2^)	4 (2.7)	2 (1.6)	2 (9.5)	0.094
Alanine aminotransferase, IU/L	74.1±98.3	72.9±103.1	81.6±62.3	0.707
Gamma glutamyltranspeptidase, IU/L	44.5±40.3	47.1±42.6	32.9±26.3	0.295
Platelet count, 10^9^/L	188±65	183±63	219±73	0.020*
Liver biopsy				
Fibrosis stage				
F2–4	127 (84.7)	113 (87.6)	14 (66.7)	0.055
F3–4	84 (56.0)	82 (63.6)	2 (9.5)	<0.001*
F4	68 (45.3)	67 (51.9)	1 (4.8)	<0.001*
Maximal activity^a^				
A2–4	132 (88.0)	113 (87.6)	19 (90.5)	0.751
A3–4	60 (40.0)	49 (38.0)	11 (52.4)	0.236
A4	18 (12.0)	14 (10.9)	4 (19.0)	0.284
Lobular activity				
A2–4	125 (83.3)	106 (82.2)	19 (90.5)	0.530
A3–4	35 (23.3)	26 (20.2)	9 (42.9)	0.047*
A4	1 (0.7)	1 (0.8)	0	0.999
Periportal activity				
A2–4	118 (78.7)	100 (77.5)	18 (85.7)	0.568
A3–4	51 (34.0)	43 (33.3)	8 (38.1)	0.804
A4	18 (12.0)	14 (10.9)	4 (19.0)	0.284
Biopsy length, cm	17.9±2.2	18.0±2.2	17.1±2.0	0.125
LSM				
LSM value, kPa	11.7±7.3	11.7±7.7	11.3±3.2	0.659
Success rate, %	96.5±8.0	96.4±8.3	97.0±6.8	0.747
IQR/M, kPa	0.14±0.09	0.14±0.09	0.13±0.10	0.830
IQR/M >0.3	10 (6.7)	8 (6.2)	2 (9.5)	0.632

Variables are expressed as mean ± SD or n (%).

Maximal activity^a^ grade was defined as the higher one between lobular and periportal activity.

BMI, body mass index; LSM, liver stiffness measurement; kPa, kilopascal; IQR/M, interquartile range/median LSM value.

*P<0.05.

### Liver histology and corresponding LSM values

The median length of LB samples was 17 (range, 15–25) mm. The fibrosis stage and maximal activity grade are summarized in **Table 2**. Most patients (*n* = 126, 84.0%) showed no steatosis (S0) and only 1 (0.1%) patient with F1 fibrosis showed moderate steatosis (S2).

**Table 2:** Liver histology and corresponding LSM values (n = 150).

Fibrosis		Maximal activity		LSM (kPa)
Stage	n (%)	Grade	n	Median (range)
1	23 (15.3)	1	6	5.2 (3.7–8.7)
		2	13	5.4 (4.3–15.3)
		3	4	7.2 (6.0–8.7)
		4	-	-
2	43 (28.7)	1	-	-
		2	11	6.8 (4.6–11.0)
		3	21	7.8 (5.3–14.3)
		4	11	8.8 (5.0–17.1)
3	16 (10.7)	1	2	10.5 (10.0–11.0)
		2	7	8.3 (5.7–9.3)
		3	1	13.9
		4	6	11 (7.9–21.8)
4	68 (45.3)	1	10	9.1 (5.3–14.1)
		2	41	13.3 (7.6–34.3)
		3	16	18.2 (9.2–48.0)
		4	1	45.7

LSM, liver stiffness measurement; kPa, kilopascal.

**Figure 1:**
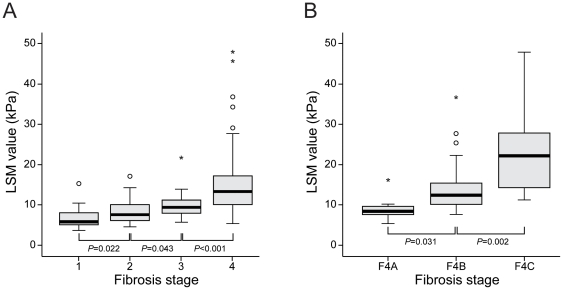
Box plots of LSM values according to fibrosis stage (A) and sub-classification of cirrhosis (B). Median LSM values increase significantly as fibrosis stage increases [6.4 kPa for F1 (range 3.7–15.3), 9.1 kPa for F2 (range 4.6–17.1), 10.0 kPa for F3 (range 5.7–21.8), and 12.0 kPa for F4 (range 5.3–48.0); all *P*<0.05] and histologic sub-classification of cirrhosis also shows a significant increment in the median LSM values [8.5 kPa for F4A (range, 5.3–16.2), 12.4 kPa for F4B (range, 7.6–37.8), and 22.2 kPa for F4C (range, 11.2–48.0); all *P*<0.05].

The median and range of LSM values according to maximal activity grade in each fibrosis stage are listed in **Table 2**. The median LSM values increased significantly as fibrosis stage increased (6.4 kPa for F1, 9.1 kPa for F2, 10.0 kPa for F3, and 12.0 kPa for F4; all *P*<0.05 between each fibrosis stage; **Figure 1(A)**) and the median LSM values tended to increase as fibrosis stage and activity grade increased (**Figure S1**).

Histological sub-classification of cirrhosis also showed a significant increase in the median LSM values (8.5 kPa for F4A, 12.4 kPa for F4B, and 22.2 kPa for F4C; all *P*<0.05 among F4A, B, and C; **Figure 1(B)**) with no significant difference in ALT levels (median 32.5 (range, 19–70) IU/L for F4A. 42 (range, 12–112) IU/L for F4B, and 42.5 (range, 25–91) IU/L for F4C; all *P*>0.05).

### Influence of necroinflammatory activity on LSM according to each fibrosis stage

**Figure 2:**
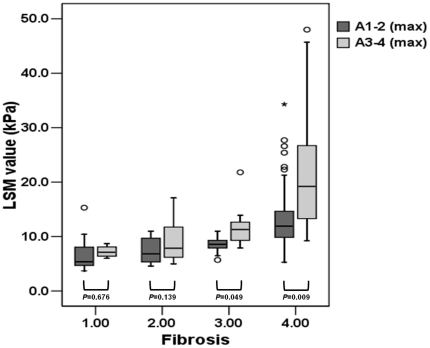
Distribution of LSM values according to fibrosis stage and maxiaml activity grade 1–2 *vs*. 3–4. The median LSM value was significantly higher in maxiaml activity grade 3–4 than 1–2 in F3 and F4 fibrosis stage (8.6 [range, 5.7–11.0] *vs*. 11.3 kPa [range, 7.9–21.8] in F3, *P* = 0.049; 11.9 [range, 5.3–34.3] *vs*. 19.2 kPa [range, 9.2–48.0] in F4, *P* = 0.009).

LSM values between maximal activity grade 1–2 versus 3–4 were compared in each fibrosis stage (**Figure 2**). The median LSM values were not significantly different between maximal activty grade 1–2 and 3–4 in F1 and F2 fibrosis stage (*P* = 0.676 and 0.139, respectively), whereas those were significantly higher in maximal activty grade 3–4 than 1–2 in F3 and F4 fibrosis stage (8.6 *vs*. 11.3 kPa in F3, *P* = 0.049; 11.9 *vs*. 19.2 kPa in F4, *P* = 0.009).

The ALT levels in F4 were 40.8±15.6 IU/L in the cases with maximal activity grade 1–2 and 60.7±23.3 IU/L in those with maximal activity grade 3–4, which were significantly different (*P* = 0.004). In contrast, the ALT levels in F1, F2, and F3 showed no significant difference between maximal activity grade 1–2 versus 3–4 (50.6±38.6 *vs*. 173.3±114.9 IU/L in F1, *P* = 0.121; 72.0±51.0 *vs*. 139.1±176.6 IU/L in F2, *P* = 0.224; 35.4±23.3 *vs*. 112.3±121.1 IU/L in F3, *P* = 0.146).

### Correlations between variables

Among the study variables, the highest correlation was noted between ALT level and maximal activity grade (correlation coefficient, 0.497; *P*<0.001), followed by a correlation between ALT and age (correlation coefficient, −0.290; *P*<0.001) and another between ALT and gender (correlation coefficient, −0.170; *P* = 0.038).

### Comparison between patients with non-discordance and those with discordance

Cutoff LSM values from our cohort were 6.0 kPa for ≥F2 (Se, 90.6%; Sp, 60.9%; PPV, 92.7%; NPV, 53.8%), 7.5 kPa for ≥F3 (Se, 96.4%; Sp, 57.6%; PPV, 74.3%; NPV, 92.7%), and 9.4 kPa for F4 (Se, 80.9%; Sp, 73.2%; PPV, 71.4%; NPV, 82.2%), giving a discordance between LSM and LB in 21 (14.0%) patients (**Table 3**). When we compared baseline characteristics between patients with non-discordance and those with discordance, age, platelet count, the proportion of F3–4 and F4, and the proportion of lobular activity grade 3–4 at the time of LB was significantly different from each other (all *P*<0.05; **Table 1**). Although 10 (6.7%) patients with IQR/M>0.3 and one (0.7%) with a success rate <60% were identified, IQR/M and success rate were not selected as significant factors associated with discordance (*P* = 0.830 and *P* = 0.747, respectively).

**Table 3:** Distribution of fibrosis stage based on LB and LSM in patients with discordance (n = 21).

	LB high group^a^	LSM high group^b^
	(n = 2, 9.5%)	(n = 19, 90.5%)
Fibrosis stage		
F1	–	7
F2	–	12
F3	1	–
F4	1	–
LSM value, kPa		
F1 (<6.0 kPa)	2	–
F2 (≥6.0 kPa)	0	–
F3 (≥7.5 kPa)	–	4
F4 (≥9.4 kPa)	–	15

LB, liver biopsy; LSM, liver stiffness measurement; kPa, kilopascal.

A discordance was defined as a discordance of at least two stages between LB and LSM.

LB high group^a^ was defined as patients with higher fibrosis stage based on LB.

LSM high group^b^ was defined as patients with higher fibrosis stage based on LSM.

### Patients with discordance between LB and LSM

When we stratified these 21 patients with discordance into two groups (LB high group, defined as patients with higher fibrosis stage based on LB, and LSM high group, defined as those with higher fibrosis stage based on LSM), only 2 (9.5%) patients were stratified into the LB high group and 19 (90.5%) into the LSM high group. The distribution of fibrosis stages based on LB and LSM is indicated in **Table 3**.

The mean maximal activity grade and ALT levels of two patients with discordance in LB high group showed a trend to be lower than those of the 129 patients with non-discordance (1.5±0.7 *vs*. 2.4±0.8, *P* = 0.150 and 25.0±12.7 *vs*. 72.9±103.1 IU/L, *P* = 0.514, respectively). The mean maximal activity grade of the 19 patients with discordance in the LSM high group was higher than the 129 patients with non-discordance with borderline statistical significance (2.7±0.9 *vs*. 2.4±0.8, *P* = 0.074), whereas ALT levels only showed a trend to be higher in the LSM high group than in the 129 patients with non-discordance (87.6±62.5 *vs*. 72.9±103.1 IU/L, *P* = 0.547).

**Figure 3:**
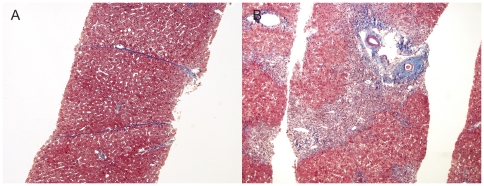
Histology of LB high group (A) and LSM high group (B) (Masson trichrome, original magnification ×100). (A) a patient in LB high group (F4A, maximal activity grade of A1, and ALT level of 34 IU/L) showed a thin fibrous septa and minimal necroinflammatory activity. (B) a patient in LSM high group (F2, maximal activity grade of A4, and ALT level of 168 IU/L) showed periportal fibrosis with bridging necrosis.

Of the two cases in the LB high group, one was classified as F4A fibrosis stage, with a maximal activity grade of A, and the other was classified as stage F3, with a maximal activity of A2. The histology of the former patient with F4A showed a thin fibrous septa with minimal necroinflammatory activity (**Figure 3(A)**). Among the 19 cases in the LSM high group, 7 and 12 cases showed F1 and F2 fibrosis stage, respectively, and their maximal activity was A1–2 in 8, A3 in 8, and A4 in 3 cases, respectively. One histological example of the LSM high group showed periportal fibrosis with bridging necrosis (**Figure 3(B)**).

### Independent predictors of discordance

In the multivariate binary logistic regression analysis, only F3–4 and F4 at the time of LB showed independent negative associations with discordance (*P* = 0.002; HR, 0.073; 95% CI, 0.014–0.390 for F3–4 and *P* = 0.014; HR, 0.067; 95% CI, 0.008–0.574 for F4). Given that IQR/M was demonstrated to be a significant predictor of discordance in previous studies [13], [15], we adjusted F3–4 and F4 using IQR/M, although IQR/M was not a significant discriminating factor of discordance in our univariate analysis. However, IQR/M was not statistically significant (*P* = 0.551 with F3–4 and *P* = 0.495 with F4), whereas F3–4 and F4 at the time of LB remained significant independently (*P* = 0.002; HR, 0.073, 95% CI, 0.014–0.387 for F3–4 and *P* = 0.013; HR, 0.065; 95% CI, 0.008–0.559 for F4; **Table 4**).

**Table 4:** Independent predictors of discordance between LSM and LB.

	Univariate	Multivariate			
	*P* value	*P* value(with F3–4)	*P* value(with F4)	Hazard ratio	95% confidence interval
Age	0.008*	0.906	0.640	-	-
Platelet count	0.020*	0.769	0.574	-	-
A3–4 (lobular)	0.047*	0.658	0.543	-	-
F3–4	<0.001*	0.002*	-	0.073	0.014–0.390
F4	<0.001*	-	0.014*	0.067	0.008–0.574
*IQR/M adjusted*					
Age	0.008*	0.851	0.596	-	-
Platelet count	0.020*	0.710	0.520	-	-
A3–4 (lobular)	0.047*	0.613	0.512	-	-
IQR/M	0.830	0.551	0.495	-	-
F3–4	<0.001*	0.002*	-	0.073	0.014–0.387
F4	<0.001*	-	0.013*	0.065	0.008–0.559

LSM, liver stiffness measurement; LB, liver biopsy; IQR/M, interquartile range/median value.

*P<0.05.

**Figure 4:**
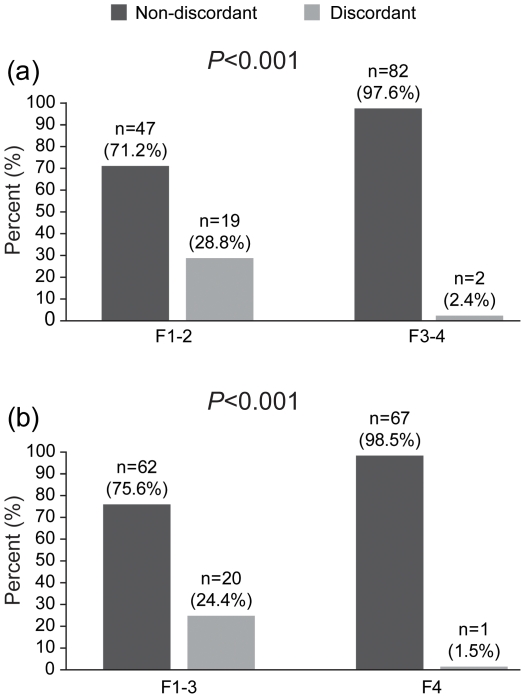
Percentage of patients with non-discordance and those with discordance in fibrosis stage F1–2 *vs*. F3–4 (A) and F1–3 *vs.* F4 (B). The estimated odds ratios of discordance were 0.060 in patients with F3–4 (*P*<0.001; 95% confidence interval [CI], 0.013–0.271) and 0.046 in those with F4 (*P*<0.001; 95% CI, 0.006–0.355).

**Figure 4** showed the discordant rate according to F3–4 and F4. The estimated odds ratios of discordance, calculated by the chi-squared test, were 0.060 in patients with F3–4 (*P*<0.001; 95% CI, 0.013–0.271) and 0.046 in those with F4 (*P*<0.001; 95% CI, 0.006–0.355).

## Discussion

Although LSM is an accurate method that evaluates the degree of liver fibrosis [5], many extrinsic factors have significant influences on LSM [6]–[10]. Additionally, LSM should satisfy the three intrinsic prerequisites of ≥10 valid measurements, success rate ≥60%, and IQR/M<0.3 to maintain the validity needed to reflect the real fibrotic state of liver [5]. However, these intrinsic prerequisites are only the manufacturer's recommendation.

As a result, several studies have investigated the influence of IQR/M on the accuracy of LSM [13]–[15]. Two studies with chronic hepatitis C (CHC) only [13] or mostly CHC [15] have proposed optimal cutoff IQR/M values of 0.21 and 0.17, respectively. In contrast, the other study with CHB did not identify IQR/M as a significant predictor of accuracy [14]. Indeed, in our study with CHB, IQR/M was not selected as a significant predictor of discordance. Reasons for this remain unclear although IQR/M in the previous CHB study [14] and ours (both mean IQR/M 0.14) was much lower than in CHC studies (0.23 [13] and 0.16 [15]) indicating that LSM performed more accurately. We believe that IQR/M may not be a sensitive marker to predict discordance in CHB because of the influence of inhomogeneous histological features or necroinflammation that overwhelm the influence of IQR/M on LSM [22]. Consistent with the literature [13]–[15], success rate was not a significant predictor of discordance in our study, which might mean that ‘high quality,’ reflected by lower IQR/M, does matter for the accurate interpretation of LSM, rather than a ‘high percentage’ of successful shots.

In addition to IQR/M, fibrosis stage (F0–2 *vs*. F3–4) and elevated ALT (>1.5–2× ULN) were proposed as significant extrinsic predictors of discordance [13]–[15]. However, controversy remains regarding fibrosis stage. Lucidarme *et al*. [13] concluded that advanced fibrosis (F3–4) was correlated with discordance, while Kim *et al*. [14] and Myers *et al*. [15] proposed minimal fibrosis (F0–2) as a significant predictor of discordance between LB and LSM. In our study, F3–4 and F4 showed a negative correlation with discordance. The significantly lower rate of discordance in F4 may be explained by an unlimited upper cutoff LSM value for cirrhosis until 75 kPa in this study design. Accordingly, F3–4, which included a high proportion of F4 (80.9%), also showed a negative correlation with discordance. From these results, we suggest that a different predictor of discordance may be produced according to a different distribution of F4. Furthermore, this hypothesis may explain why ALT level was not selected as a significant predictor of discordance in our study with a higher prevalence of F3–4 (56.0%) than in a previous study (20.3%) that proposed ALT as a significant predictor of discordance [15]. This confounding effect of elevated ALT may have been attenuated in our study due to a higher proportion of F4, which is free to misdiagnosis due to LSM overestimation by elevated ALT, despite similar ALT levels between the two studies [mean 61 IU/L [15] *vs*. 74 IU/L in the present study].

Thus, the potentially masked influence of necroinflammation in F4, the consistent reports on the overestimating effects of elevated ALT, and the significant correlation between lobular activity grade 3–4 and discordance in our univariate analysis prompted us to investigate further the effects of necroinflammatory activity grade on LSM. The maximal activity grade 3–4 significantly influenced LSM values in F3 and F4, but not in F1 or F2, which may be explained in several ways. First, the mean ALT level was not significantly different in A1–2 versus A3–4 in F1 and F2 in our cohort. Thus, the ALT effect could not be revealed. Second, extrinsic factors, such as liver congestion [7] and respiration [23] resulting in a change of portal flow concurrent with necroinflammation, may have influenced the performance of LSM when liver fibrosis was insufficient (≤F2) to be detected by LSM. These combined effects of several confounders might have concealed the effects of necroinflammation on LSM, consistent with a recent meta-analysis on LSM reporting the relatively lower performance of LSM to predict significant fibrosis (≥F2) [24].

Most patients (90.5%) with discordance were stratified into the LSM high group, indicating that LSM values were subject to overestimation due to necroinflammation or high ALT. Although statistical significance for necroinflammation and high ALT was not seen when we compared patients with non-discordance with those in the LSM high group, the clinical implications should be further investigated in future studies, considering the borderline statistical significance of high ALT (*P* = 0.074) and the small sample size of the LB high group. Indeed, clinical variables, such as ALT level, which showed the best correlation with necroinflammatory activity in our study, are needed to predict the discordance ‘before LB,’ because histological variables, such as fibrosis stage or activity grade, which are only available ‘after LB’ are not helpful for the prediction of the discordance between LB and LSM. Indeed, the concept of excluding subjects with high ALT to enhance the accuracy of LSM has already been proposed [17], [25]. Although ALT was not a significant predictor, the optimal cutoff ALT level to predict discordance was 55 IU/L (data not shown). Furthermore, the mean ALTs of maximal activity grade 3–4 in F3 and F4 that raised LSM values significantly were 60.7 and 112.3 IU/L, respectively. Because the ALT cutoff seemed to be around 1.5–3× ULN in our study and 1.5–2× ULN in previous ones [14], [15], [26], ALT level ≤3× ULN may be optimal to enhance LSM performance.

Interestingly, LSM values showed a significant stepwise increment according to F4A, B, and C without a significant difference in ALT levels in our study, indicating that LSM can further stratify patients with cirrhosis. Thus, mild liver cirrhosis (F4A) might have a higher chance of being underestimated by LSM than moderate or severe cirrhosis (F4B or F4C), especially when necroinflammatory activity or ALT level is low. This could be a reason why one patient with F4A and A1 activity grade belonged to the LB high group. Although the histological sub-classification of cirrhosis is gaining clinical relevance [27], stratification of cirrhosis according to the Laennec system or LSM should be further validated via long-term follow-up studies using solid clinical end-points, such as liver-related death or development of hepatocellular carcinoma.

In conclusion, advanced fibrosis stage (F3–4) or cirrhosis (F4) showed a negative correlation with discordance between LB and LSM in patients with CHB, and maximal activity grade 3–4 significantly influenced LSM values in F3–4 and F4. Thus, future studies should investigate how to control for the clinical marker of ALT, which may bridge histological information to enhance the accuracy of LSM.

## Supporting Information

Figure S1 **The median LSM values according to fibrosis stage and activity grade [maximal (A), lobular (B), and periportal activity grade (C)].** The median LSM values tended to increase as fibrosis stage and activity grade increase.
(TIF)
