# Supplementary figures and images for: Discordance between Liver Biopsy and FibroScan® in Assessing Liver Fibrosis in Chronic Hepatitis B: Risk Factors and Influence of Necroinflammation
Source: PLoS One. 2012 Feb 23;7(2):e32233. doi: 10.1371/journal.pone.0032233 (PMC3285687; doi:10.1371/journal.pone.0032233)

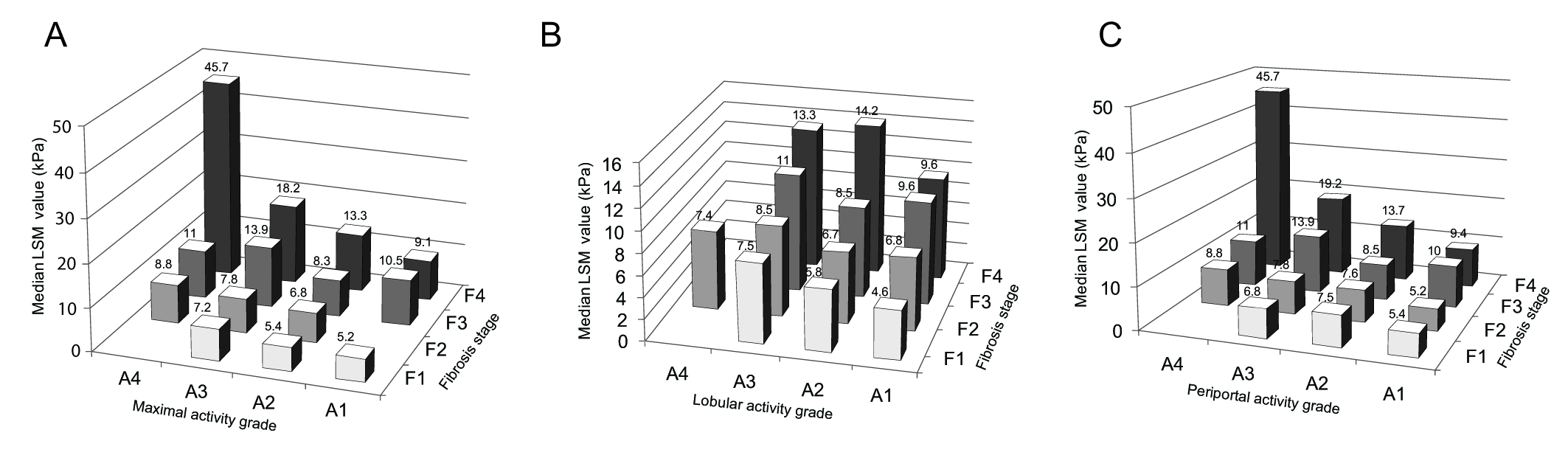

Supplement: Figure S1 — The median LSM values according to fibrosis stage and activity grade [maximal (A), lobular (B), and periportal activity grade (C)]. The median LSM values tended to increase as fibrosis stage and activity grade increase. (TIF) [file pone.0032233.s001.tif]
